# Dentinal tubule penetration of a bioceramic sealer after different final irrigants: SEM and CLSM study

**DOI:** 10.6026/973206300221773

**Published:** 2026-03-31

**Authors:** Rahul Vaswani, Pallav Patni, Swadhin Raghuwanshi, Sanket Pandey, Shubham Tripathi, Dharika Patel

**Affiliations:** 1 Department of Conservative Dentistry and Endodontics, Sri Aurobindo College of Dentistry, Indore, Madhya Pradesh, India; 2 Department of Conservative Dentistry and Endodontics, Government Dental College and Hospital, Jamnagar, Gujarat, India; 3 Department of Oral Health, College of Dentistry and Dental Clinics, University of Iowa, Iowa USA

**Keywords:** Confocal laser scanning microscope, dentinal tubules, energy-dispersive x-ray spectroscopy, scanning electron microscope (SEM), smear layer

## Abstract

The extent of bioceramic sealer penetration into dentinal tubules following root canal obturation remains insufficiently validated using combined analyses on the same sample with scanning electron microscopy, confocal laser scanning microscopy and ion nano-complex evaluation. Therefore, it is of interest to investigate the extent of penetration of a bioceramic sealer into dentinal tubules after the use of various final irrigants and evaluated ion nano-complex formation. Mandibular incisor teeth were instrumented, divided into four groups based on the final irrigation protocol and obturated with gutta-percha and a bioceramic sealer tagged with a fluorescent dye (Fluo-3), followed by evaluation using confocal laser scanning microscopy and scanning electron microscopy with energy-dispersive X-ray spectroscopy. Although statistical analysis did not reveal significant differences among the groups, Twin Kleen demonstrated superior performance in multiple comparisons. The HEBP component of Twin Kleen, due to its compatibility with sodium hypochlorite and its ability to reduce debris accumulation and smear layer formation, may contribute to enhanced sealer penetration.

## Background:

The prime goal of root canal therapy is to totally remove the microbes from the tooth and to avoid recontamination. While root canal instrumentation reduces the bacterial load, it is still nearly impossible to completely disinfect the canals due to their complex anatomy [1]. While chemo-mechanical debridement is a common approach, it's challenging to completely cleanse and debride the root canal system [2]. Incorporating irrigation is crucial alongside instrumentation, as it effectively eliminates necrotic tissue and microbes, including troublesome smear layers [3]. Previous research has demonstrated that the occurrence of the smear layer can hinder the sealer's dispersion into the dentinal tubules, thereby shielding bacteria within these tubules [4]. EDTA, MTAD, REDTA, citric acid (CA), phosphoric acid, maleic acid (MA) and their combinations have long used as chelating agents for eliminating this smear layer [5]. The effective seal following biomechanical procedures is crucial for averting microbes from gathering and causing reinfection in the root canal-treated tooth. Since the sealers fill the irregularities within the root canal system, the extent of penetration into the tubules is critical, which directly impacts the effectiveness of the root canal seal [6]. Enhanced contact in the interphase of dentin and sealer, achieved through increased sealer penetration, is closely linked to improved sealability [7]. Furthermore, sealer penetration has the potential to exert a germicidal effect within the tubules, which becomes more pronounced as proximity to microbes' increases [8]. Over time, researchers have used scanning electron microscopes (SEMs) to assess how well sealers can penetrate dentinal tubules. Utilizing SEM produces images that enable a thorough inspection of the dispersion of the sealer in the tooth root [9]. The Energy-dispersive X-ray Spectroscopy (EDX) microanalysis highlights various ions vital role in the effectiveness of sealer materials for periapical repair. Materials rich in calcium content are likely to yield improved filling and periapical repair outcomes. Moreover, the application of calcium silicate cement holds promise for enhancing biological activity and promoting barrier formation, offering the potential for enhanced periapical repair results in dental procedures [10]. Confocal laser scanning microscopy (CLSM) offers a comprehensive way to understand the sealer distribution and presence along the entire perimeter of the root in the dentin. This can be achieved at relatively low magnifications, typically at 100x, using fluorescent rhodamine-marked sealers [11, 12]. While the Fluo-3 begins as a non-fluorescent compound, when it joins with calcium, it begins to give fluorescence. For the bioceramic sealers based on their calcium-silicate ingredients, this enables them to connect with Fluo-3 dye, inducing their fluorescence [13]. It should be noted that Fluo-3 fluorescence is not detected by the Ca^2+^ ions of dentin, which guarantees that the results reflect the amount of Ca^2+^ ions in the sealer [14]. Therefore, it is of interest to study of dentinal tubule penetration of a bioceramic sealer after different final irrigants.

## Materials and Methods:

Eighty human permanent mandibular incisors with completely formed apices were selected. These teeth were shortened to 16 mm in length from the apex to the crown using a diamond disk under water. Hand instrumentation till #20k file (Kerr USA) and added instrumentation was done by ProTaper gold rotary files till F2 size (Dentsply Maillefer, Ballaigues, Switzerland). Throughout the instrumentation, 5.25% NaOCl (Neelkanth, Jodhpur, India) was used for irrigation and flushed with 10 mL of deionized water. 30-gauge irrigation needles were used for irrigating all the root canals (Max-I-Probe; Dentsply Maillefer).

## For the final irrigation, 4 groups were divided:

[1] Group 1- 37% Phosphoric acid (Dental Avenue, Pune, India)

[2] Group 2- 17% EDTA Solution (Dental Avenue, Pune, India)

[3] Group 3- Twin Kleen with 3% NaOCl (MAARC, Maharashtra, India)

[4] Group 4 - Control Group treated with Saline

The introduction of the final irrigant needle was 1 mm less than the working length. Afterward, the canals were rinsed with distilled water. Root canals were filled with the ceraseaeal-B bioceramic sealer (Maarc, Maharashtra, India) coated over to the GP along with 1 mg Fluo-3 penta ammonium salt indicator (Life Technologies, Carlsbad, CA) with a single cone technique. Following obturation, Cavit (3M ESPE, St Paul, MN) was filled on the access cavities and samples were kept at 37°C and 100% humidity for 2 weeks. Afterward, each treatment group sample was sliced into apical, middle and coronal sections (2, 5 and 8 mm from the apex, respectively) and polished through silicon carbide abrasive papers. CLSM imaging was performed by Leica TCS-SPE confocal microscope (Leica, Mannheim, Germany) and images were analyzed. FV10-ASW 4.2 software was used to import each image of the sample taken and outline areas where sealer penetrated the dentinal tubules for measurement purposes Figure 1 (see PDF). The extent of sealer penetration was measured from the canal wall to the furthest point of infiltration within the dentin. Following this, the SEM analysis was done after overnight drying. The root sections were affixed to aluminum stubs and subjected to sputter-coating with a 10% gold-palladium alloy using a sputtering machine (JFC-1100E, Ion Sputtering Device). This process was performed to render the otherwise non-conductive samples electrically conductive, thereby preventing the occurrence of imaging artifacts that commonly arise when non-conductive samples are analyzed under electron microscopy, as they are prone to charging from the electron beam. The specimens were subsequently examined using a scanning electron microscope (JEOL JSM-840A, Tokyo, Japan) at an accelerating voltage of 8-10 kV and a resolution of 2 nm. The entire surface, along with specific regions (apical, middle and coronal), was analyzed at magnifications ranging from x12 to x2000. Elemental composition and distribution were assessed through Energy Dispersive X-ray (EDX) analysis, utilizing the NSS Spectral Analysis System 2.3 (Thermo Fisher Scientific Inc., Suwanee, GA). EDX measurements were performed with an electron beam spot size of less than 50 nm, an accelerating voltage of 25 kV and a beam current of 110 mA, in accordance with the manufacturer's specifications. Spectra were collected over a 100-second live time period. This technique generates X-rays based on the elemental composition of the sample, providing the data for the analysis of the sealer's penetration depth into the dentin and the identification of compounds formed.

## Results and Discussion:

The Kruskal-Wallis analysis was done and the results were statistically significant, as evidenced by p-values of 0.01 for all comparisons, indicating meaningful differences in performance across the solutions and depths tested. A suggestively reduced maximum depth of sealer penetration was seen with the Wilcoxon signed rank in the apical area when compared to the coronal area Table 1 (see PDF). Among all the groups, Twin Kleen with 3% NaOCl emerged as the most effective solution. At a depth of 2mm, it had an average effectiveness of 477.50±80.64 Figure 2 (see PDF), which increased to 1137.50±165.07 at 8mm Figure 3 (see PDF). This trend demonstrates its superior capacity to maintain and enhance performance in depth. The second most effective solution was 17% EDTA, which also showed significant effectiveness of penetration against the 37% phosphoric acid and saline, particularly at greater depths. At 2mm, it had an average effectiveness of 399.50±79.96, which increased to 1020.50±137.82 at 8mm. Following 17% EDTA, 37% phosphoric acid demonstrated moderate effectiveness. Its performance improved with depth, from 337.50±71.80 at 2mm to 926.50±105.64 at 8mm. This suggests that it can be a viable option for applications where moderate depth efficacy is required. On the lower end of the effectiveness spectrum was saline. Saline had the lowest effectiveness values at all depths-197.00±31.97 at 2mm and 582.50±68.43 at 8mm. This group demonstrated the least capability in achieving the desired outcomes, making it the least effective option in this study. All the groups effective penetration was summarized in Figure 4 (see PDF). The endodontic sealer's tubular penetration is inherent to the chemical and physical properties it holds [15]. The sealer displays maximum penetration in dentin at the coronal third, with penetration gradually decreasing as we move towards the apex. The dentin present at the apical third is defined as poorly permeable and sclerotic, with fewer tubules relative to the coronal thirds, which could be a reason behind this pattern [16]. The Bio-C Sealers fluidity, hydrophilicity and minor particle size allow for greater penetration into the dentin [17]. The tags of sealer inside the tubules have shown that Bio-C Sealer had a consistent penetration in the dentin. Its hydrophilic nature and the nanometric particle structure allow for deeper and more homogeneous penetration [18]. Various methods can be used to measure dentinal tubule penetration depth, including SEM, light microscopy and CLSM. SEM helps in the visualization of dentinal tubules with high magnification and accurate penetration depth measurement [19].

CLSM is non-destructive microscopy and also produces fewer artifacts, as it doesn't need any distinct specimen processing and allows determining the overall dispersion into dentinal tubules [20]. The persistence of microbes within the canals has mainly remained the reason behind endodontic failure due to inadequate shaping, cleansing and sealing. In case of improperly sealing the end of the root canal, tissue fluids can enter and provide a breeding ground for bacteria, leading to treatment failure [21]. The Rhodamine B dye is known for its strong attraction to moisture and weaker affinity for calcium within sealer compositions. This has raised concerns about the possibility of it separating from or leaching out of its blend with the sealer [22]. This could potentially lead to the detection of even minor levels of moistness in dentin and default fluorescent emission from the sealer, even its deeper infiltration into tubules might mislead the results; thus, it is suggested by recent researchers to use Fluo-3 dye as a fluorophore indicator to study how much the sealer has travelled into dentine tubules [10]. Using a dye like Fluo-3 indicator mixed with a calcium silicate-based sealer can be used to evaluate the degree of sealer penetration, as it binds to the calcium in the sealer and becomes fluorescent. This allows for a more accurate assessment of sealer penetration using CLSM [23]. When it comes to removing debris from the canal, using a combination of EDTA and NaOCl can be effective. However, it's important to use EDTA before obturation, as using it during cleaning can lead to erosion of the dentin. Additionally, prolonged usage of EDTA or a combination of NaOCl and EDTA can further erode the dentin [24]. In a recent study, it was found that the Twin Clean application had a significant impact on the depth of sealer penetration by removing the smear layer [25]. Surface roughness is among the key factors in the biocompatibility of any substance, as it has a direct effect on cell adhesion to its surface. A former study proved that all sealers tested showed irregular microsized particle clustering, having sizes between 0.5 µm and >200 µm, mainly containing the zirconium and silica particles [10]. On the other hand, Ceraseal demonstrates smaller round-shaped particles clustered in a matrix seen among layers ranging from 0.3-68µm [26]. Root canal sealers that give smooth surfaces are thought to have better cell adhesion. Nonetheless, surface regularity does not act by itself on cell adhesion and biocompatibility, as it is other parameters like chemical composition that can impact these processes. In addition, this might act on the bacteria already present with the possible antibacterial action of chemical constituents of the sealers [27]. Elemental analysis using EDX by Root Canal Sealers by Seux (1991) demonstrated that fibronectin binds to calcite crystals and this aids in the adhesion and differentiation of cells throughout time, eventually leading to increased numbers of observed hard tissue. Hence, materials containing calcium have a high potential for complete filling. On the other hand, when a sealer has considerably lower calcium levels, its ability to induce periapical healing may not be as predicted in this study [28]. Calcium hydroxide is then widely used as a periapical repair inducer and to make a mineralized root apex during the tooth canal insulation phase of treatment. However, because of its increased calcium and hydroxyl ion content as well as some type of mechanical barrier (matrix), it may provide a protective effect against overfilling when used. Previous studies showed that calcium silicate cement produces an appetite layer on the surface after interaction with body fluids and helps in the activation/differentiation of cells at the bone-periapical interface, in this way accelerating biological activity [29]. Moreover, zirconium was substantially present in CeraSeal compared to the other MTA-containing sealers. Zirconia with proper radiopacity, as well as no deleterious effect with the hydration process of the sealer, has become an excellent option [30]. Zirconium oxide may not be substantially more biocompatible than bismuth oxide; both agents easily brushed off scales, but zirconium teeth were far less affected by discoloration. Zirconium oxide at controlled concentrations was reported to enhance the radiopacity of tricalcium silicate cement without affecting their setting [31]. Previous studies have indicated that imaging a similar sample using both CLSM and SEM is not ideal, where the CLSM dye may interfere with the coating or conduction processes during SEM analysis [32]. However, after conducting our study, we found that using CLSM followed by SEM with the Fluo-3 dye enabled us to successfully capture imaging data. The study's SEM results showed that the sealer's nano-complex can chemically change the dentin's surface and subsurface by creating an ion-rich covering. This alteration may strengthen the dentin's mechanical strength in teeth with root fillings and increase the interfacial integrity between the dentin and sealer. According to elemental analysis, there were also indications that the sealer and its ion complexes penetrated the dentin, where the Zirconium ion was found in abundance at over 5% (Figure 2, 3 - see PDF).

## Conclusion:

We report data for the varying effectiveness of different solutions at multiple depths, guiding the selection of the most appropriate solutions for specific application needs. Twin Kleen with 3% NaOCl, given its consistent and superior performance, is highly recommended. The 17% EDTA and 37% phosphoric acid also present themselves as strong alternatives for the final irrigation. Data shows the importance of choosing the right final irrigant to achieve optimal sealing in endodontic treatments.

## Advancement to knowledge:

This study provides insights into the dentinal tubule penetration of a bioceramic-based sealer following different final irrigation protocols. The combined use of Confocal Laser Scanning Microscopy (CLSM), Scanning Electron Microscopy (SEM) and Energy-dispersive X-ray Spectroscopy (EDX) on the same samples offers a comprehensive evaluation of sealer penetration and ion nano-complex formation at the dentin-sealer interface. The findings may help clinician's select appropriate irrigation protocols to enhance sealer penetration and improve the effectiveness of root canal therapy.
